# Diarrhea deaths in children among countries with different levels of the human development index

**DOI:** 10.1016/j.dib.2018.02.019

**Published:** 2018-02-15

**Authors:** Mina Riahi, Ali Akbar Mohammadi, Vahid Kazemi Moghadam, Zahra Sadat Robati, Mohammad Bidkhori

**Affiliations:** a Department of Health, Shahrekord University of Medical Science, Shahrekord, Iran; b Department of Environmental Health Engineering, Neyshabur University of Medical Sciences, Neyshabur, Iran; c Student Research Committee, Neyshabur University of Medical Sciences, Neyshabur, Iran; d Department of Public Health, Neyshabur University of Medical Sciences, Neyshabur, Iran

**Keywords:** Diarrhea, Drinking water, Sanitation, Human development index

## Abstract

The present study investigated the effect of human development index (HDI) on diarrheal deaths per 1000 live births in children under 5 years old in 2015. In addition, the association between HDI, and the use of improved drinking-water sources and sanitation facilities were evaluated in this year. 75 countries that their information was available in Global Analysis and Assessment of Sanitation and Drinking-Water (GLAAS) conducted by the World Health Organization (WHO) were included in this study. The data required was obtained from WHO and United Nations Development Programme (UNDP) websites. Pearson's correlation coefficient and linear regression were used to evaluate the correlation and association between the variables, respectively. The results showed that there is a significant relationship between HDI and diarrhea-associated deaths per 1000 live births in children during 2015 (*B* = −354.85, CI95%: −408.91, −300.79). In addition, HDI was associated with the use of improved drinking-water sources (*B* = 83.93, CI95%: 64.71, 103.15) and improved sanitation facilities (*B* = 199.90, CI95%: 174.39, 225.42) in 2015. These findings indicate the association between HDI and the measures relevant to diarrheal disease among children. Therefore, in order to achieve to the Millennium Development Goals regarding child health, policymakers should concentrate on environmental and social factors affecting health.

**Specifications table**

**Table:** 

Subject area	environmental science
More specific subject area	Nursing and health Professions
Type of data	Table and figure
How data was acquired	Secondary data
Data format	Raw and analyzed
Experimental factors	In order to determine the association between the variables, linear regression and Pearson's correlation analyses were performed by STATA 14.
Experimental features	Investigation relationship between human development index (HDI) whit diarrhea deaths per 1000 live births in children under 5 years and Use of improved drinking-water sources و Use of improved sanitation facilities
Data source location	Data Obtained from: WHO, UNDP
Data accessibility	Data are available from:
	World Health Organization. UN-Water Global Analysis and Assessment of Sanitation and Drinking-Water (GLAAS) 2017 report 2017 [cited 2017 November 8, 2017]. Available from: http://www.who.int/water_sanitation_health/publications/glaas-report-2017/en/.
	World Health Organization. Distribution of causes of death among children aged <5 years, Diarrhoeal diseases 2016 [cited 2017 October 23, 2017]. Available from: http://apps.who.int/gho/data/view.main.ghe2002015-CH3?lang=en
	United Nations Development Programme. Human Development Data (1990–2015) 2015 [cited 2017 December 13, 2017]. Available from: http://hdr.undp.org/en/data.

**Value of the data**

• It is necessary to understand the factors affecting death in children under five years old.
• The results showed that efforts should be concentrated on environmental and social factors in order to achieve Millennium Development Goals for child health.
• This study provides an analysis to the status of countries in relation to child mortality and access to improved drinking-water sources and sanitation facilities based on the country's human development index.

## 1 Data

The data required for the analyses included diarrhea-associated deaths per 1000 live births in children, the use of improved drinking-water sources and improved sanitation facilities (at national, urban and rural levels) in percentage term in 2015 and HDI in 2015 (Table 1).

**Table 1:** Diarrhea-associated diarrhea deaths per 1000 live births in children under 5 years, the use of improved drinking-water sources and improved sanitation facilities (at national, urban and rural levels) in percentage term, and HDI in 2015.

**Country**	**Sanitation rural**	**Sanitation urban**	**Sanitation national**	**Water rural**	**Water urban**	**Water nation**	**Diarrhea Deaths**	**HDI**
**Afghanistan**	27	45	32	47	78	55	11.5	.479
**Albania**	90	95	93	95	95	95	0.2	.764
**Argentina**	98	96	96	100	99	99	0.2	.827
**Azerbaijan**	87	92	89	78	95	87	1.8	.759
**Bangladesh**	62	58	61	87	87	87	2.3	.579
**Barbados**	96	96	96	100	100	100		.795
**Belarus**	95	94	94	99	100	100		.796
**Bhutan**	33	78	50	100	100	100	2.1	.607
**Bolivia**	28	61	50	76	97	90	2.4	.674
**Bosnia and Herzegovina**	92	99	95	100	100	100		.75
**Botswana**	43	79	63	92	99	96	2.7	.698
**Brazil**	52	88	83	87	100	98	0.4	.754
**Burkina Faso**	7	50	20	76	97	82	7.2	.402
**Burundi**	49	44	48	74	91	76	8	.404
**Cambodia**	30	88	42	69	100	76	1.9	.563
**Chile**	91	100	99	93	100	99		.847
**China**	64	87	76	93	98	95	0.3	.738
**Colombia**	68	85	81	74	97	91	0.3	.727
**Costa Rica**	92	95	95	92	100	98	0.1	.776
**Côte d'Ivoire**	10	33	22	69	93	82	6.9	.474
**Cuba**	89	94	93	90	96	95	0.1	.775
**Dominican Republic**	76	86	84	82	85	85	1.5	.726
**Ecuador**	81	87	85	76	93	87	0.9	.739
**El Salvador**	60	82	75	87	97	94	0.9	.68
**Ethiopia**	28	27	28	49	93	57	4.9	.448
**Fiji**	88	93	91	91	100	96	0.9	.736
**Georgia**	76	95	86	100	100	100	0.1	.769
**Ghana**	9	20	15	84	93	89	4.1	.579
**Guatemala**	49	78	64	87	98	93	2.1	.64
**Guinea**	12	34	20	67	93	77	7.7	.424
**Haiti**	19	34	28	48	65	58	7.2	.493
**Honduras**	78	87	83	84	97	91	1.6	.625
**India**	28	63	40	93	97	94	4.7	.624
**Jamaica**	84	80	82	89	97	94	0.3	.73
**Kenya**	30	31	30	57	82	63	3.6	.555
**Kyrgyzstan**	96	89	93	86	97	90	1.1	.664
**Lao**	56	94	71	69	86	76	7.6	.586
**Lesotho**	28	37	30	77	95	82	8.6	.497
**Liberia**	6	28	17	63	89	76	6.2	.427
**Lithuania**	83	97	92	90	100	97		.848
**Madagascar**	9	18	12	35	82	52	4.5	.512
**Malaysia**	96	96	96	93	100	98	0.1	.789
**Maldives**	98	97	98	98	100	99	0.2	.701
**Mali**	16	38	25	64	97	77	10.8	.442
**Mexico**	74	88	85	92	97	96	0.4	.762
**Micronesia**	49	85	57	87	95	89	2.2	.638
**Mongolia**	43	66	60	59	66	64	1.4	.735
**Mozambique**	10	42	21	37	81	51	6.9	.418
**Nepal**	43	56	46	92	91	92	2.1	.558
**Nigeria**	25	33	29	57	81	69	11	.527
**Pakistan**	51	83	64	90	94	91	7.3	.55
**Panama**	58	84	75	89	98	95	0.9	.788
**Papua New Guinea**	13	56	19	33	88	40	3.9	.516
**Paraguay**	78	95	89	95	100	98	1.2	.693
**Peru**	53	82	76	69	91	87	0.8	.74
**Philippines**	71	78	74	90	94	92	2.2	.682
**Rwanda**	63	59	62	72	87	76	3.1	.498
**Senegal**	34	65	48	67	93	79	4.1	.494
**Serbia**	94	98	96	99	99	99		.776
**Solomon Islands**	15	81	30	77	93	81	2	.515
**South Africa**	61	70	66	81	100	93	3.6	.666
**Swaziland**	56	63	57	69	94	74	6.3	.541
**Tajikistan**	95	94	95	67	93	74	3.4	.627
**Thailand**	96	90	93	98	98	98	0.4	.74
**Timor-Leste**	27	69	41	61	95	72	4.8	.606
**Tonga**	89	98	91	100	100	100	0.6	.721
**Ukraine**	93	97	96	98	96	96	0.2	.743
**Tanzania**	8	31	16	46	77	56	3.9	.531
**Uruguay**	93	97	96	94	100	100	0.1	.795
**Uzbekistan**	100	100	100		98		2.3	.701
**Vanuatu**	55	65	58	93	99	94	3.9	.597
**Venezuela**	70	97	94	78	95	93	0.8	.767
**Viet Nam**	70	94	78	97	99	98	1.4	.683
**Zambia**	36	56	44	51	86	65	5.6	.579
**Zimbabwe**	31	49	37	67	97	77	6.7	.516

### 1.1 Correlation between HDI and diarrhea deaths in children under 5 years

**Fig. 1:**
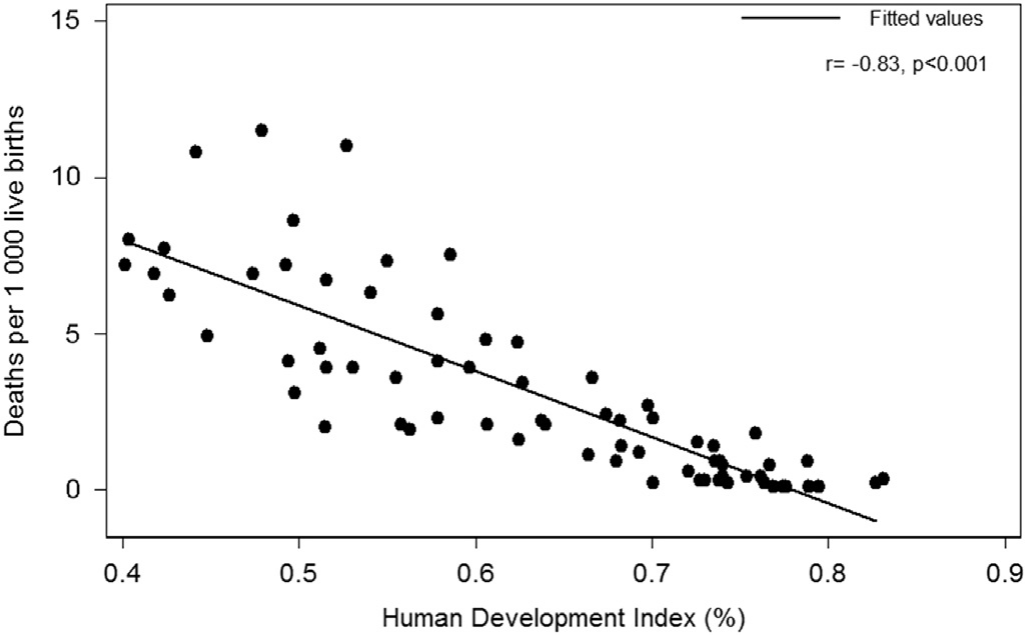
Correlation between HDI and diarrhea deaths per 1000 live births in children under 5 years in 2015.

The results showed that HDI in 2015 had a significant negative correlation with diarrhea-associated deaths per 1000 live births (*r* = −0.83, *p* =<0.001). As it can be seen in Fig. 1, diarrhea-associated deaths are reduced by increasing HDI.

### 1.2 Correlation between HDI and use of improved drinking-water sources

**Fig. 2:**
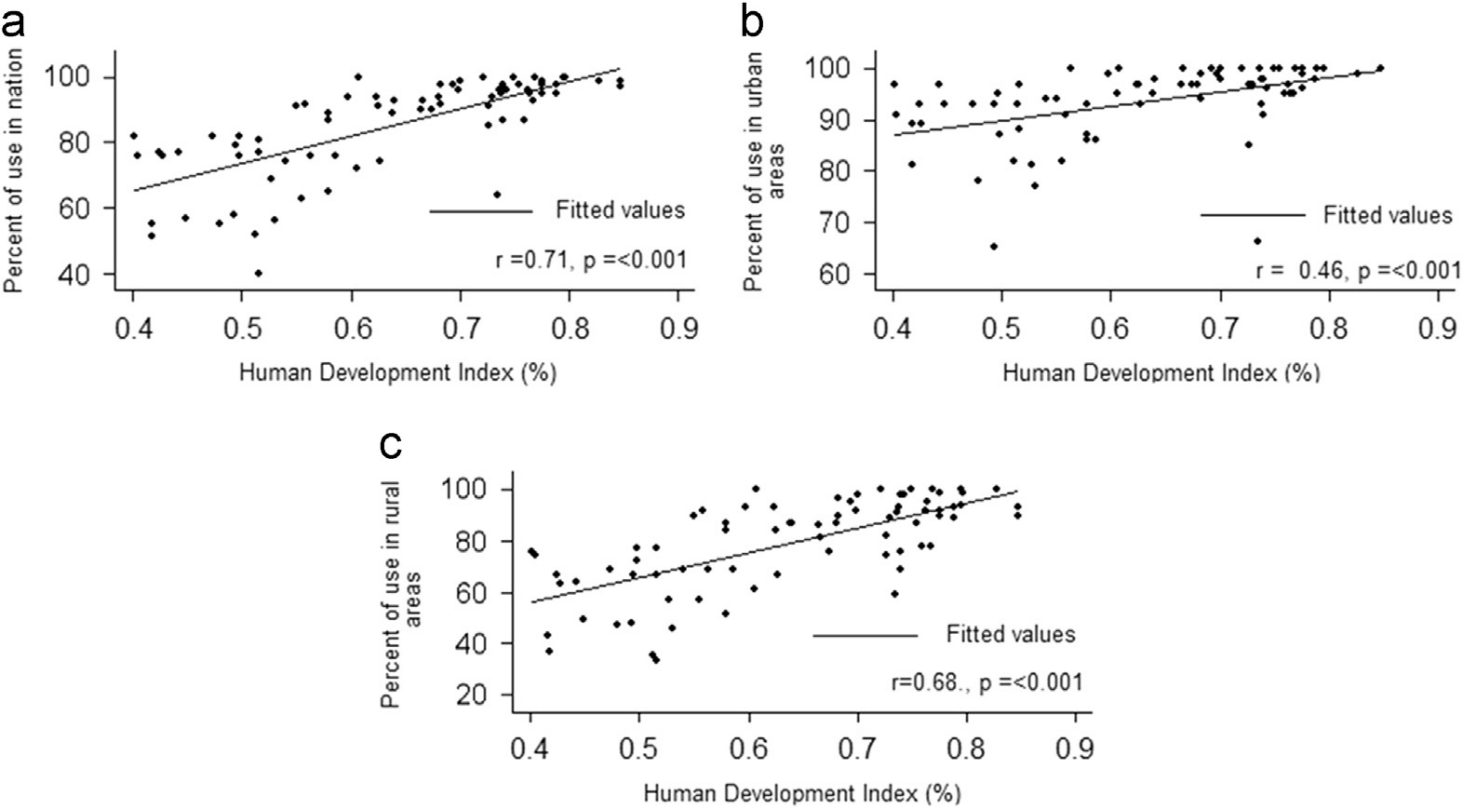
Correlation between HDI and Use of improved drinking-water sources in 2015 in 3 levels: a: Correlation in national levels, b: Correlation in urban areas and c: Correlation in rural areas.

According to Fig. 2, a significant positive correlation was observed between HDI in 2015 and the use of improved drinking-water sources at the national level (*r* = 0.71, *p* =< 0.001). In addition, HDI had positive correlations with the use of improved drinking-water sources in urban areas (=0.46, *p* =< 0.001), as well as rural level (*r* = 0.68, *p* =< 0.001).

### 1.3 Correlation between HDI and use of improved sanitation facilities

**Fig. 3:**
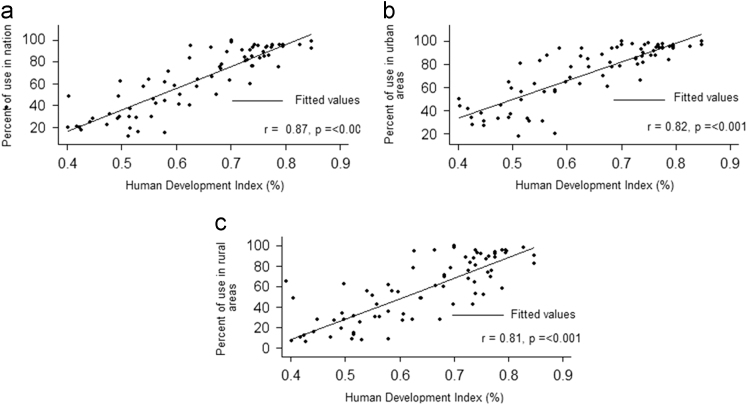
Correlation between HDI and use of improved sanitation facilities in 2015 in 3 levels: a: Correlation in national levels, b: Correlation in urban areas and c: Correlation in rural areas.

The correlation between HDI in 2015 and improved sanitation facilities at the national (*r* = 0.87, *p* =< 0.001), urban (*r* = 0.82, *p* =< 0.001), and rural (*r* = 0.81, *p* =< 0.001) levels were statistically significant (Fig. 3).

### 1.4 Linear regression analysis

Linear regression analysis was used to determine the relationship between the variables. According to Table 2, HDI had a significant relationship with diarrhea-associated deaths, and the use of improved drinking-water sources and improved sanitation facilities at national, urban and rural levels. On average with a one-unit increase in HDI, diarrhea-associated deaths decrease, while the use of improved drinking-water sources and improved sanitation facilities increases. The effect of HDI on the use of improved sanitation is greater than the use of improved drinking-water sources. In fact, people's access to improved sanitation is more dependent on the HDI and development status.

**Table 2:** Effect of HDI on diarrhea deaths, use of improved drinking-water sources and Use of improved sanitation facilities.

Independent variable	Dependent variable	B	*P*-value	95% Confidence Interval
HDI	**Water**			
	nation	83.93	< 0.001	(64.71, 103.15)
	urban	28.26	< 0.001	(15.77 ,40.76)
	rural	96.61	< 0.001	(72.32, 120.91)
				
HDI	**facilities**			
	national	199.90	< 0.001	(174.39, 225.42)
	urban	162.88	< 0.001	(137.26, 188.51)
	rural	200.25	< 0.001	(167.32,233.18)
				
HDI	**Diarrhea deaths**	−21.12	< 0.001	(−24.53, −17.70)

## 2 Experimental design, materials and methods

### 2.1 Study countries description

Diarrheal diseases are one of the major causes of death in children [1], [2], [3], [4]. 75 countries that their information was available in the Global Analysis and Assessment of Sanitation and Drinking-Water (GLAAS) were included in this study. The data about diarrhea-associated deaths per 1000 live births in children under 5 years, use of improved drinking-water sources (at the national, urban and rural level), and use of improved sanitation facilities (at the national, urban and rural level) were acquired from WHO website [5], [6]. In addition, HDI values were obtained from UNDP website [7]. Human development index is combined of three parts, including life expectancy at birth, mean years of schooling, and gross national income per capita and its value is between 0 and 1 [8], [9], [10], [11].

### 2.2 Analytical procedures

Pearson's correlation coefficient was used to calculate the correlation between the variables. Linear regression was used to analyze the relationship between the variables. All the statistical analysis were performed using STATA 14.
